# Social Information and Economic Decision-Making in the Ultimatum Game

**DOI:** 10.3389/fnins.2012.00103

**Published:** 2012-07-06

**Authors:** Celia Gaertig, Anna Moser, Sonia Alguacil, María Ruz

**Affiliations:** ^1^Department of Experimental Psychology, University of GranadaGranada, Spain; ^2^Laboratory for Biological and Personality Psychology, Department of Psychology, University of FreiburgFreiburg, Germany; ^3^Department of Cognitive, Perceptual and Brain Sciences, University College LondonLondon, UK

**Keywords:** ultimatum game, decision-making, social information, uncertainty

## Abstract

The present study tested how social information about the proposer biases responders’ choices of accepting or rejecting real monetary offers in a classic ultimatum game (UG) and whether this impact is heightened by the uncertainty of the context. Participants in our study conducted a one-shot UG in which their responses had direct consequences on how much money they earned. We used trait-valenced words to provide information about the proposers’ personal characteristics. The results show higher acceptance rates for offers preceded by positive words than for those preceded by negative words. In addition, the impact of this information was higher in the uncertain than in the certain context. This suggests that when deciding whether or not to take money from someone, people take into account what they know about the person they are interacting with. Such non-rational bias is stronger in an uncertain context.

## Introduction

Within the emerging field of judgment and decision-making, it is broadly accepted that humans are not purely rational decision-makers (see Camerer, 2003). A recent line of research within this field tries to capture the nature of decision-making in social contexts. This is particularly interesting as many of our everyday choices involve or affect other people.

Regarding the aspects that influence such decisions, different studies stress the importance of emotions as a biasing factor. It has been shown that displayed positive and negative facial expressions (e.g., Scharlemann et al., 2001; Ruz and Tudela, 2011) as well as induced emotions unrelated to the task (Harlé and Sanfey, 2007) influence decision-making in inter-personal interactions. Further aspects that have been found to influence decision-making include the physical attractiveness and the gender of people with whom we interact (e.g., Solnick and Schweitzer, 1999; Solnick, 2001; Eckel and Grossman, 2008).

As another clear biasing factor, social information has been shown to have an effect on economic choices in social contexts with high degrees of uncertainty, such as those in the Trust Game (Delgado et al., 2005). In an iterated Trust Game, trading partners described as morally praiseworthy were trusted more often than those with neutral or untrustworthy moral character, even when the descriptions had no predictive value regarding the actual behavior of the partners. As the reciprocity of the unknown partner in this game has direct consequences on the monetary outcome of the truster, it seems useful to rely on any relevant information available to guide trust choices. This matches previous data in non-social contexts showing that uncertainty increases the value of information. For example, people might be more disposed to being influenced when they lack complete knowledge of the situation (e.g., Behrens et al., 2007; Rushworth and Behrens, 2008) and might try to make use of any additional piece of information they can gather. As a practical example, when making decisions on the stock market, investors facing unstable prices are more receptive to new tips than during stable periods (Schachter et al., 1985).

There are other social situations in which the degree of uncertainty is smaller than in the Trust Game, such as when making choices of accepting or rejecting offers in the Ultimatum Game (UG; Güth et al., 1982). In this game two people interact to divide a sum of money between them. One of them, the *proposer*, receives a certain amount of money. He has to split it into two parts, one for him and one for his counterpart, the *responder*. The responder then can either accept or reject the proposal. If he accepts it, both receive their part; if he rejects it, neither the proposer nor the responder gets any payoff. For the responder the degree of uncertainty of this situation is low, seeing that he reacts to a given decision of the proposer in form of a monetary offer.

From the economic point of view, the self-interested, income-maximizing *homo economicus* should accept every kind of offer, no matter how little it is (Nash, 1950). However, such predicted behavior is not confirmed in experimental settings where small offers (of 20% or below of the initial amount) are rejected about half of the times (Camerer, 2003). Irrational rejection of unfair offers in the UG may be explained by several factors, such as inequity aversion (Fehr and Schmidt, 1999) and emotions accompanying the perception of unfairness. Responders often feel wounded pride and anger when facing unfair offers and tend to punish their selfish game partner favoring emotional satisfaction over money gains (Pillutla and Murnighan, 1996). Physiological (van’t Wout et al., 2006) and neuroimaging studies (Sanfey et al., 2003) support the important role of emotions in the UG, showing, for example, that emotionally relevant brain regions, such as the right anterior insula, are activated when participants are faced with unfair offers (Sanfey et al., 2003).

As the responder in the UG finds himself in a situation where the decision of the proposer has already taken place, there is no obvious reason why his choices should change depending on the information he has about the person he interacts with. However, even in such a certain context social information about the proposer seems to influence decision-making.

Using a modified version of the UG, Ruz et al. (2011) showed that personal descriptions of game partners biased decisions to the same set of offers. Offers preceded by negative words were rejected with higher probability than those preceded by positive words. In addition, rejection responses were faster after negative words, whereas acceptances were faster following positive words, which suggests that the social information primed action tendencies. Thus, even though the words did in no manner predict how fair the following offer was going to be, social information regarding the partner affected the decisions of participants in this game. Furthermore, these authors introduced a manipulation of the uncertainty of the social situation and found that the social bias had a much larger effect when the context of the game was uncertain. Some characteristics of this study, however, limit the scope of the results. First, Ruz et al. (2011) used a modification of the UG instead of the original task setting. In their version, the difference between the two parts of a split was either one (fair offers) or four (unfair offers) and the responder’s part of the split could be either higher or lower than their partner’s amount. Thus, offers could be either convenient or inconvenient for the participant. Furthermore, to enable measurement of response times, they required participants to take their decision within a time limit of 1500 ms. When participants did not respond on time, they saw a message stating that the higher amount of the split would be added to their partner’s earnings. This leaves open the question of whether similar results would be obtained in a version closer to the classic UG. Additionally, they did not pay real money to participants, and thus it could be claimed that the social information biased responses because participants did not have anything at stake.

Two recent studies solve part of these problems. Campanhã et al. (2011) used the classic UG and demonstrated that friendship with the proposer modulated the choices made by the responder in the game. More specifically, responders rejected unfair offers less frequently when the proposer was believed to be a friend rather than an unknown person. However, as several rounds were played with the same partner, it is not sure that the responders’ choices reflected responses to a single offer instead of bargaining behavior. It must also be noted that no real money was offered to participants. Furthermore, interactions with a friend can always be affected by the long-term relation we hold with this person, which may have affected the results found by Campanhã et al. (2011).

Another study (Marchetti et al., 2011) showed that the type of information about the proposer provided to the responder has an influence on decision-making in the UG. Most interestingly, they found an interaction between the psychological description of the partner and the fairness of the offer: a negative (selfish) description of the proposer led to a decreased acceptance rate of fair offers, while a positive (generous) description led to an increased acceptance rate of unfair offers. As this study employed one-shot interactions with unknown partners, concerns regarding long-term interactions or even friendship do not arise. However, as in the game by Ruz et al. (2011) and in the study of Campanhã et al. (2011), participants in this study did not receive money in accordance with their decisions.

Thus, it still has to be tested whether social information regarding the partner in a classic UG biases people’s decisions to offers of real money, which was the goal of the present study. We conducted a classic computerized, one-shot UG and used trait-valenced words to describe the moral characteristics of otherwise unknown partners. As previous results indicate that the level of uncertainty modulates the scope of the biasing information in a modified UG (Ruz et al., 2011), we included a manipulation of uncertainty to explore whether the level of uncertainty also affects responses in the classic UG.

Participants of our experiment played the role of the responders and received either fair or unfair offers from several different proposers represented by the computer. Following the findings of the classic UG it was hypothesized that more fair than unfair offers would be accepted. As a description of the partners’ characteristics in each trial, each offer was preceded by a word with positive or negative valence highly linked to morality and trustworthiness (see Table 1). It was hypothesized that acceptance rates would be biased by this social information, with higher acceptance rates for offers preceded by positive than for those preceded by negative words. Additionally, participants had either full or incomplete information about the outcome of their choices, which modulated the uncertainty of the game. In the certain condition, participants were informed of which part of the split corresponded to them, while this information was not given in the uncertain condition. It was hypothesized that the influence of the valence of the word would be higher in the uncertain context. Experiment 1 confirmed these hypotheses. Experiment 2 showed that personal information only influenced choices when it was *attributed* to the partners in the game.

**Table 1:** **List of words selected as stimuli in the study and acceptance rates of offers depending on the word that preceded the offer**.

Positive words	Accepted offers (%)	Negative words	Accepted offers (%)
Friend	79 (19)	Hostile	60 (25)
Humble	76 (19)	Selfish	53 (26)
Honorable	75 (18)	Guilty	53 (25)
Generous	74 (17)	Disloyal	52 (27)
Loyal	73 (19)	False	48 (28)
Warm	73 (19)	Cruel	47 (29)
Honest	72 (23)	Traitor	47 (27)
Kind	71 (20)	Criminal	41 (29)
Mean	74 (16)	Mean	50 (23)

*Standard deviations are given in parentheses*.

## Experiment 1

Experiment 1 manipulated the level of uncertainty (uncertain vs. certain), the type of offer proposed by the partner in the trial (fair vs. unfair) and the valence of the word preceding the offer (positive vs. negative).

### Materials and methods

#### Participants

Thirty-six native Spanish-speaking, right-handed students from the University of Granada participated in the study (23 female, 18–27 years, average 21.5). All participants signed a consent form approved by the Department of Experimental Psychology of the University of Granada. In exchange for their participation in the study, participants were paid. The payment amount depended on their earnings during the game task and ranged from about 3–6 Euros.

#### Stimuli

Sixteen trait-valenced words were selected from the Spanish translation of the Affective Norms English Word database (ANEW; Redondo et al., 2007) as stimuli in the study. The words selected provided moral and trustworthiness information and had either a positive (in average 7.5, *SE* = 0.5) or a negative valence (in average 1.9, *SE* = 0.3). Positive and negative words were equated regarding number of letters and frequency of use (average number of letters: 6; average frequency: 21.72; all *p*s > 0.390). The English translation of the words used is listed in Table 1.

#### Procedure

First, an introduction explaining the rules of the UG was given to the participants. They were told that they were going to play the UG in the role of the responder and were going to receive offers that other participants had made in previous experiments of the lab. To enhance the plausibility of this cover story, participants completed a short questionnaire in which they themselves generated offers for 16 anonymous partners. For each partner they were asked to decide how to divide 10 Euros into two parts, one for them and the other one for their partner.

Before conducting the game, participants were informed that they were playing with actual money, which was to strengthen their motivation to make real decisions. They were informed that one point earned in the game was to be exchanged for 1.5 cents of Euro. To avoid possible influences of previous reciprocation, they were told that on each trial they were going to play with a different partner, who was never the same across the game.

The initial amount of the proposer was always 10 Euros, and the split proposed to the responder was presented in the middle of the screen. To every participant, the same set of splits including two kinds of fair offers (5/5, 4/6) and three different kinds of unfair offers (1/9, 2/8, 3/7) was presented. These types of offers match the range of offers that humans normally propose in the role of the proposers in the UG.

In total, participants received 128 offers and they had to accept or reject each of them by pressing the number 1 or 2 (counterbalanced across participants) of the computer keyboard. If they accepted the offer, their part of the split was added to their earnings and their partner for the trial received the other part. If they rejected the offer, no transaction was carried out.

Additionally, each offer was preceded by a word. Participants were told that the words represented personal descriptions of their partner and that these characteristics had been obtained through questionnaires completed by the same participants that made the offer proposals in previous experiments. The same words were presented in a different random order to each participant. Half of the words had a positive valence and the other half had a negative one (see Table 1). In reality, the valence of the words was not related in any way to the type of offer, as each word was followed equally often by fair and unfair offers.

To test whether the uncertainty of the context influenced decision-making in the classic version of the UG, the whole task consisted of two blocks (order counterbalanced across participants) differing in the information provided to participants. In the *certain* block, as in the original game, participants knew which part of the split would be added to their earnings if they accepted the offer. Therefore, for each split the two numbers were presented in different colors (green and red) and participants were told which of the colors corresponded to them (counterbalanced across participants). Accordingly to the range of offers normally proposed by humans in the game, the participants’ part of the split always consisted in the smaller or equal (5/5 offer) number. In the *uncertain* block, in contrast, participants did not know which part of the split would be added to their earnings, as both numbers were presented in black. Therefore they lacked that part of the information.

**Figure 1:**
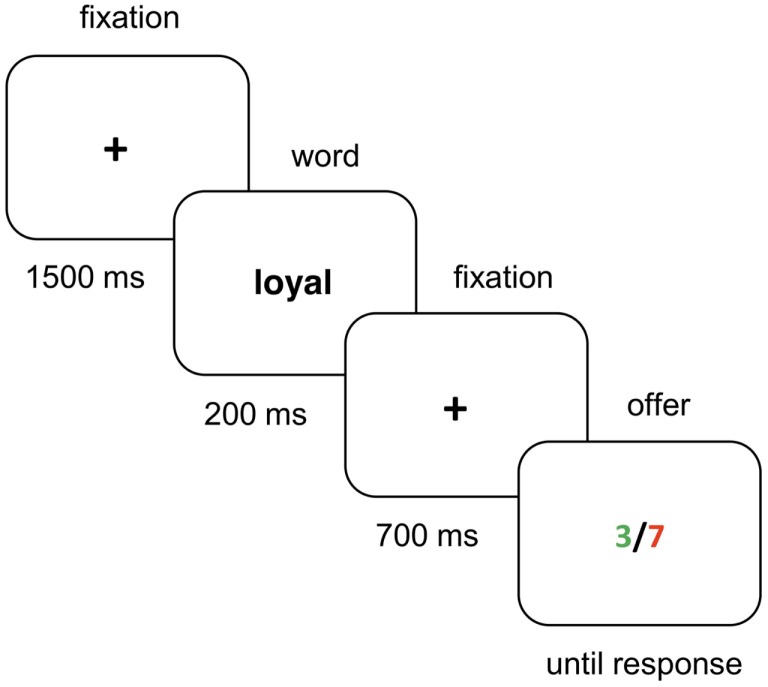
**Sequence of events in a trial**.

The experiment was conducted using a PC running E-Prime software (Schneider et al., 2002). Each trial (see Figure 1) started with a fixation cross presented for 1500 ms (+ ; 0.4°) in the center of the screen. Following this, the word (average 2.5°) was displayed for 200 ms, and then, the fixation point was presented for another 700 ms. Subsequently, the offer (1.5°), consisting of two numbers separated by a slash symbol, appeared in the center of the screen until the participant made the response. Following the decision of the participant, the next trial began. The whole experiment consisted of 128 trials and had an approximate duration of 10 min.

### Results

The acceptance rate measured in percentage of accepted offers was analyzed by a 2 (uncertainty: uncertain vs. certain) × 2 (offer: fair vs. unfair) × 2 (valence of the word: positive vs. negative) multifactorial ANOVA.

**Figure 2:**
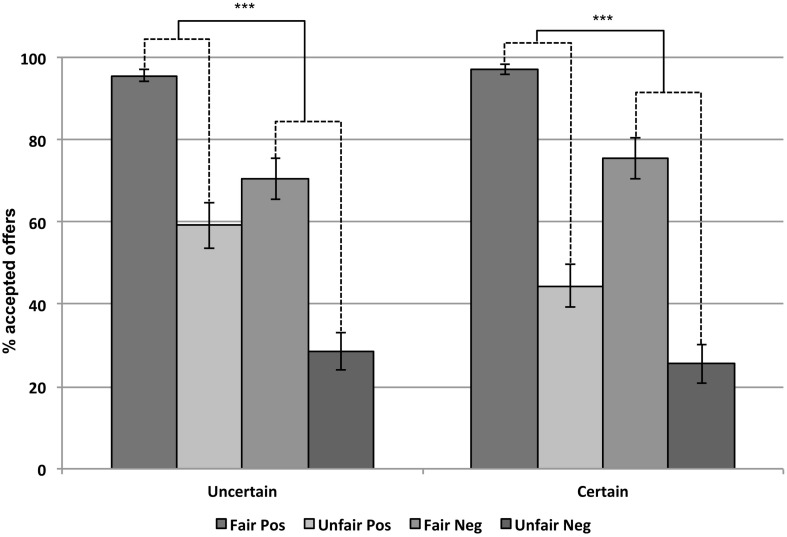
**Acceptance rates of fair and unfair offers preceded by positive and negative words in the uncertain and certain condition (Experiment 1)**. Error bars represent standard errors of the mean. Lines represent significant effects of the positive vs. negative valence of the trait-words (***p < 0.001).

On average participants accepted 62.1% of all offers across the experiment (Figure 2). There was a main effect of the fairness of the offer (*F*_1,35_ = 86.25, *p* < 0.001), as fair offers were accepted more often (*M* = 85%, *SE* = 17%) than unfair ones (*M* = 39%, *SE* = 25%). Decisions were also influenced by the valence of the word (*F*_1,35_ = 33.64, *p* < 0.001). Offers were accepted more often when they were preceded by positive words (*M* = 74%, *SE* = 16%) than by negative words (*M* = 50%, *SE* = 23%). Table 1 shows the acceptance rates of offers separately for each trait-valenced word.

In addition, a significant interaction between uncertainty and the fairness of the offer was found (*F*_1,35_ = 12.38, *p* < 0.01). In the certain condition, the effect of the offer was higher (51%) than in the uncertain condition (39%). Furthermore, unfair offers were accepted more often in the uncertain (44%) than in the certain context (35%; *F*_1,35_ = 9.65, *p* < 0.01), whereas there was no significance difference between the acceptance rate of fair offers in both contexts (83 vs. 86%, *F*_1,35_ = 3.13, *p* = 0.086). Finally, as predicted there was a significant interaction between uncertainty and valence of the word (*F*_1,35_ = 8.01, *p* < 0.01). The effect of the valence of the word was higher in the uncertain (28%) than in the certain condition (20%).

In the analysis described above all offers are included (5/5, 4/6, 3/7, 2/8, and 1/9) for both certain and uncertain contexts. The experiment included the fair offer 5/5 to mimic the range of offers normally proposed in the classic UG. Nevertheless, it is clear that when facing this offer participants knew that they would get five points, both in the certain and uncertain blocks. Therefore, we conducted a second analysis excluding the 5/5 offers to ensure that our main results were not affected by this. This analysis replicated all previous results.

### Discussion

The results of Experiment 1 confirm our initial hypotheses. First of all and in agreement with typical findings in the UG (Camerer, 2003), the fairness of the offer influenced participants’ choices in a major way as fair offers were accepted more often than unfair offers.

With regard to the main question of interest of this study, our results show that trait-valenced words influence decision-making in the classic UG in which the decisions of participants influenced how much money they earned. Offers preceded by positive trait-words were accepted more often than those preceded by negative trait-words. Crucially, we show that this bias exists in a classic UG in which long-term effects were not present, as participants were interacting only once with a partner not personally known to them. Furthermore, participants in our study knew that their aggregate decisions of each trial determined the amount of money that they were going to earn. Therefore, they were motivated to take their decisions seriously. However, their choices of accepting or rejecting an offer were influenced by the information they had about their respective partners on each trial. Thus, they did not act purely rational, but took into account what they knew about the other person to accept or not the money. The social information biased choices, although objectively trait-valenced words and offers were not associated, insofar as positive and negative words were paired with the same set of offers. Due to such lack of association between words and offers, a learning effect can be excluded.

Two aspects might have influenced participants’ reactions in our study using the classic UG. First, it is possible that the subjective perception of fairness for all kinds of offers was biased by the social information provided. Offers made by a negatively described person might have been perceived as less fair than those made by a positively described person. Research on the UG suggests that responders in the UG reject unfair offers due to the perceived unfairness and the negative emotions arising from this perception (Pillutla and Murnighan, 1996; Sanfey et al., 2003). When providing a negative description, responders’ attention may have focused on the negative aspects of the offer (e.g., the proposer assigns more to himself than to me) rather than on the possible gains, as they were already expecting an unfair offer. Additional to and in congruence with biased subjective perception, negative emotional reactions may have been stronger in this condition, which would have led to lower acceptance rates. In the future, it could be useful to include emotional measures such as skin conductance, to directly evaluate the role that emotional reactions may play in the current paradigm.

Another accompanying explanation places the bias in a later stage of decision-making. From this perspective, offers would be perceived in the same manner, and the biasing effect would take place afterward, during the decision stage to punish the partner or not. Participants punished partners tied to a negative description more than those associated to positive characteristics. This explanation is consistent with real life experience, as people normally behave more nobly toward friendly persons, even when the chances of meeting the same person again are unlikely. We will take a closer look at the question of how to decide between these two approaches in the general discussion.

In addition to the offer fairness and the valence of the words, we manipulated the certainty of the context (uncertain vs. certain) as a third independent variable. In the uncertain context responders lacked the information about which part of the offer was assigned to them. Therefore, they could not judge whether the offer was advantageous for them or not, which is of particular relevance in the face of unequal splits.

We found that offer fairness interacted with uncertainty, as unfair offers were accepted more frequently in the uncertain than in the certain context, while the acceptance rate of fair offers did not differ in both conditions. The higher acceptance rate of unfair offers in the uncertain condition was not predicted. However, the limited possibility of objectively judging offers as convenient or inconvenient and thus, a possible lower arousal of negative emotions in the face of unfair offers, might explain this effect (see also Pillutla and Murnighan, 1996). It is also possible, that in the uncertain context responders simply feared the rejection of a convenient split and therefore accepted more offers consisting of an unequal split.

Finally and as predicted, uncertainty modulated the weight of the social information. The influence of the words was not restricted to uncertain situations, but it was higher in this context. As responders were not able to judge the convenience of the offer in the uncertain context, they might have weighted the information they received more highly and used it to generate expectations about the offer.

The results clearly show that the valence of the information about the proposer influences decisions made by participants in the classic UG. However, an alternative and less appealing explanation of our results could be that the presentation of positive and negative words primed participants with a valence-consistent mood in an automatic manner. To rule out that this is the case in our findings, we conducted Experiment 2.

A possible control experiment to study the automatic effect that valenced words may have on acceptance rates would be to use non-trait-words as primes, matched in valence and arousal ratings to the words used in Experiment 1. However, such a control would entail a change both in the words and the *instructions* (as words can no longer be attributed to the personal characteristics of the partners), which would make the interpretation of the results difficult. An alternative option, which is the one we chose for our control study, is to use exactly the same items but to change the instructions regarding the social meaning of the words in the game.

## Experiment 2

We conducted a second experiment to rule out the possibility that the impact of the words in Experiment 1 could be explained by an “automatic” priming effect driven by the mere presentation of words with high positive and negative connotations.

In Experiment 2 we told participants that the computer presented the words at random before each offer, and thus that they had nothing to do with the person who initially proposed the offer. Except for this minor change in the whole set of instructions, the experimental design was exactly the same as in Experiment 1. It was hypothesized that if the mere presence of the words, regardless of their association to the partners in the game, generates priming, results from Experiment 2 should be quite similar to those of Experiment 1. In contrast, if the key manipulation is the association of the words with the personality characteristics of the people we interact with, decision-making should not be influenced by words in the current experiment.

### Materials and methods

#### Participants

Thirty-six native Spanish-speaking students from the University of Granada participated in the study (27 female, 18–38 years, average 23.3). All participants signed a consent form approved by the Department of Experimental Psychology of the University of Granada. In exchange for their participation in the study, participants were paid. The payment amount depended on their earnings during the game task and ranged from about 3–6 Euros.

#### Stimuli and procedure

Stimuli and Methods were the same as in Experiment 1 with the exception that participants received a different instruction regarding the meaning of the words preceding each offer. They were told that the words were randomly presented by the computer program and that they were in no manner linked neither to the offers, nor to their partners in the game. As in Experiment 1, we manipulated the variables uncertainty (uncertain vs. certain), offer (fair vs. unfair), and valence of the word (positive vs. negative).

### Results

**Figure 3:**
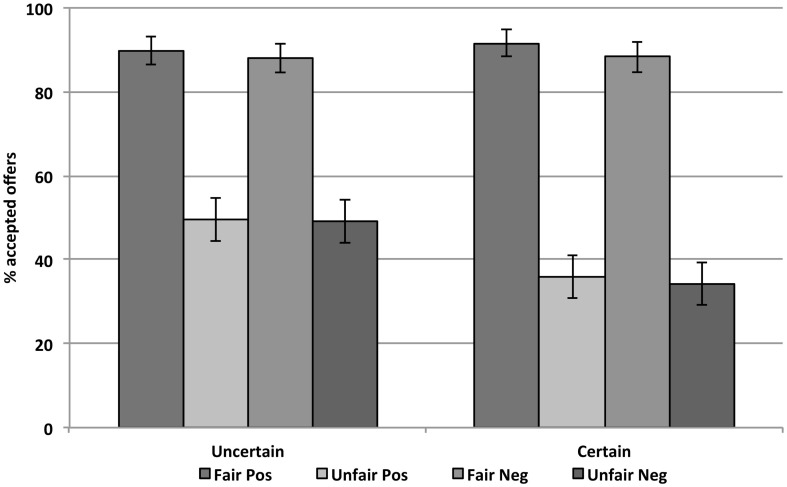
**Acceptance rates of fair and unfair offers preceded by positive and negative words in the uncertain and certain condition (Experiment 2)**. Error bars represent standard errors of the mean.

On average participants accepted 65.8% of all offers across the experiment (Figure 3). There was a significant main effect of offer fairness (*F*_1,35_ = 67.84, *p* < 0.001), as fair offers were accepted more often (*M* = 89%, *SE* = 18%) than unfair ones (*M* = 42%, *SE* = 28%). The uncertainty of the context also influenced participants’ choices. Offers in the uncertain context were accepted more often (*M* = 69%, *SE* = 16%) than in the certain context (*M* = 63%, *SE* = 18%), *F*_1,35_ = 8.82, *p* < 0.05. Furthermore, there was a significant interaction between these two variables (*F*_1,35_ = 11.13, *p* < 0.01). The effect of the offer in the certain condition was higher (55%) than in the uncertain condition (40%). Again, whereas the acceptance rate of fair offers did not differ significantly in both contexts (89 vs. 90%, *F*_1,35_ = 0.14, *p* = 0.713), unfair offers were accepted more often in the uncertain (49%) than in the certain context (35%; *F*_1,35_ = 16.38, *p* < 0.001). There was neither a significant main effect of the valence of the word, nor any other interaction (all *p*s > 0.28).

### Discussion

As it was the case in Experiment 1, in Experiment 2 participants’ choices were influenced by the fairness of the offer. In contrast to Experiment 1, however, participants’ choices were not affected by the words and there was no interaction between block uncertainty and word-valence. Hence, the mere presentation of valenced words does not prime action tendencies that lead participants to modify their acceptance decisions. This result strongly suggests that the key element for such biasing to occur is the link of the words to social characteristics of the partners in the game.

It might be argued that given that the instructions clearly told participants that the words were not related with the subsequent offers they did not pay attention to this information, which would explain the lack of effect of the words on acceptance decisions. Withdrawing attentional resources is indeed a normal consequence of deeming something as irrelevant to the task at hand (e.g., Driver, 2001), and thus it is likely that this took place in our experiment. Future studies should test the level of processing accrued by irrelevant words in this procedure by means of both explicit and more implicit memory tests (e.g., Ruz and Fuentes, 2009) as well as with brain imaging techniques (e.g., Ruz et al., 2005).

## General Discussion

The aim of the present study was to determine the influence of social information about people with whom we interact in a classic UG and to test whether such impact was modulated by the uncertainty of the context. We showed that positive and negative trait-words influenced acceptance rates to the same set of offers in a one-shot UG with unknown partners in which participants earned money, and that this effect was higher in the uncertain context. As Experiment 2 showed, the key aspect for the influence of social information was the link between trait-valenced words and the characteristics of the proposer.

Our results extend the findings of the study of Ruz et al. (2011), which used a modified version of the UG instead of the original game. Responders in our current experiment received a single offer from different anonymous partners and thus no long-term strategies can explain their behavior, as it might be the case when playing several rounds of the UG with the same partner or when interacting with a friend (Campanhã et al., 2011). As participants accumulated money with each accepted offer, their final payment depended directly on their choices in the game. Showing that, nevertheless, social information and offer fairness influenced acceptance rates of such monetary offers, the present study nicely complements recent reports using the UG without money payment (Campanhã et al., 2011; Marchetti et al., 2011; Ruz et al., 2011). Furthermore and in addition to previous studies, the manipulation of uncertainty allowed us to test how this variable modulates the impact of the social information.

It could be argued, however, that the payment associated to each offer was too small, as one point earned in the game was exchanged for 1.5 cents. Given the small amount participants were able to earn with each accepted offer, they still might not have been motivated to take the decisions seriously, and this could have led them to take social information into consideration. Perhaps, if the outcome had been higher they might have weighted their own and the others’ outcome in each trial more. However, previous studies using the UG showed that this explanation is unlikely, as it is commonly found that raising the stakes to a large amount has only a weak impact on rejection rates (Camerer, 2003). Although it is always possible to claim that bigger amounts of money could obliterate a positive result, our results show that by using payments within common ranges used in the UG we can prove that people take into account their impressions of others to accept or reject their monetary offers.

Other limits regarding the experimental setting nevertheless persist. To assure an adequate control, the experiment was conducted in the laboratory and monetary offers were presented through the computer. In addition, every participant interacted only once with each partner to avoid effects of reciprocity. One of the drawbacks of this artificiality is the caution it imposes regarding the generality of the effects to less artificial, daily life situations. Note, however, that our design replicates basic phenomena found in many previous studies, such as the rejection of unfair offers even when they are beneficial to the participants in economic terms.

On the other side, the experimental setting of the current study provides clear benefits for the design of a research study to explore the neural mechanisms underlying the biasing effect of social information. Previous studies employing the ERP methodology (Boksem and De Cremer, 2010; Campanhã et al., 2011) suggest that fair and unfair offers are perceived differently, and that this effect takes place at a relatively early stage of processing. Campanhã et al.’s (2011) ERP results further indicate that the medial frontal negativity responds to social distance, as its polarity is reversed when the offer is made by a close friend rather than an unknown proposer. In the future, it would be interesting to further explore the biological basis of inter-personal decisions and to analyze how the inclusion of social information modulates these mechanisms. To date, it remains unclear whether the effects are driven by a rather automatic kind of processing, which leads to a different perception of the offer after the presentation of valenced information, or by a controlled process at a later stage of decision-making. Further studies using electrophysiological methods could provide a closer look on the cognitive processes occurring when perceiving the offer, dependent on the valence of the social information, and, thus, could help exploring at which level of information processing social information affects decision-making.

Additional research is also needed to explore the relation between the influence of social information on responders’ choices and a possible expectancy or framing effect. Sanfey (2009) showed that expectations of fairness have a strong influence on responders’ decisions in the UG. The importance of framing effects is also discussed in Marchetti et al. (2011) to explain that positive and negative descriptions of the proposer bias the acceptance of offers in the UG. This study used psychological attributes very closely linked to fairness expectations (generous vs. selfish). Our results complement their findings, showing that different positive and negative descriptions of the partner in the UG influence decision-making. This influence exists although objectively the design of the task does not associate the trait-valenced words with the offers, as positive and negative items are presented equally often with fair and unfair offers. Future studies should test expectation generation more directly to help fully understand the relation between social information and a framing effect.

Other future lines of research could include the combination of an emotional introduction and the presentation of social information. While induced negative emotions are correlated with higher rejection rates of unfair offers (Harlé and Sanfey, 2007), in the present study the same effect was shown regarding negative social information. It would be interesting to link both approaches, testing whether the introduction of positive or negative moods biases the impact of social information.

Overall, our results show once again that human behavior is motivated by more than pure income maximization. The opinion we hold regarding the moral characteristics of people with whom we interact modulates our tendencies to accept or reject the money that they offer us. The uncertainty of the context furthermore has an effect on how we make use of this information. The present study provides further insights into the complex decision-making processes during inter-personal interactions and gives way to new questions for future research using economic games.

## Conflict of Interest Statement

The authors declare that the research was conducted in the absence of any commercial or financial relationships that could be construed as a potential conflict of interest.

## References

[B1] Behrens T. E. Woolrich M. W. Walton M. E. Rushworth M. F. (2007). Learning the value of information in an uncertain world. Nat. Neurosci. 10, 1214–1221 10.1038/nn1954 17676057

[B2] Boksem M. A. S. De Cremer D. (2010). Fairness concerns predict medial frontal negativity amplitude in ultimatum bargaining. Soc. Neurosci. 5, 118–128 10.1080/17470910903202666 19851940

[B3] Camerer C. F. (2003). Behavioral Game Theory: Experiments in Strategic Interaction. Princeton: Princeton University Press

[B4] Campanhã C. Minati L. Fregni F. Boggio P. S. (2011). Responding to unfair offers made by a friend: neuroelectrical activity changes in the anterior medial prefrontal cortex. J. Neurosci. 31, 15569–15574 10.1523/JNEUROSCI.1253-11.2011 22031902 PMC6703509

[B5] Delgado M. R. Frank R. H. Phelps E. A. (2005). Perceptions of moral character modulate the neural systems of reward during the trust game. Nat. Neurosci. 8, 1611–1618 10.1038/nn1575 16222226

[B6] Driver J. (2001). A selective review of selective attention research from the past century. Br. J. Psychol. 92(Pt 1), 53–78 10.1348/000712601162103 11802865

[B7] Eckel C. C. Grossman P. J. (2008). “Differences in the economic decisions of men and women: experimental evidence,” in Handbook of Experimental Economics Results, Vol. 1, eds Plott C. Smith V. (New York: Elsevier), 509–519

[B8] Fehr E. Schmidt K. M. (1999). A theory of fairness, competition, and cooperation. Q. J. Econ. 114, 817–868 10.1162/003355399556151

[B9] Güth W. Schmittberger R. Schwarze B. (1982). An experimental analysis of ultimatum bargaining. J. Econ. Behav. Organ. 3, 367–388 10.1016/0167-2681(82)90011-7

[B10] Harlé K. M. Sanfey A. G. (2007). Incidental sadness biases social economic decisions in the ultimatum game. Emotion 7, 876–881 10.1037/1528-3542.7.4.876 18039057

[B11] Marchetti A. Castelli I. Harlé K. M. Sanfey A. G. (2011). Expectations and outcome: the role of proposer features in the ultimatum game. J. Econ. Psychol. 32, 446–449 10.1016/j.joep.2011.03.009

[B12] Nash J. F. (1950). The bargaining problem. Econometrica 18, 155–162 10.2307/1907266

[B13] Pillutla M. M. Murnighan J. K. (1996). Unfairness, anger, and spite: emotional rejections of ultimatum offers. Organ. Behav. Hum. Decis. Process. 68, 208–224 10.1006/obhd.1996.0100

[B14] Redondo J. Fraga I. Padrón I. Comesaña M. (2007). The Spanish adaptation of ANEW (affective norms for English words). Behav. Res. Methods 39, 600–605 10.3758/BF03193031 17958173

[B15] Rushworth M. F. Behrens T. E. (2008). Choice, uncertainty and value in prefrontal and cingulate cortex. Nat. Neurosci. 11, 389–397 10.1038/nn2066 18368045

[B16] Ruz M. Fuentes L. J. (2009). Beyond perception: testing for implicit conceptual traces in high-load tasks. Conscious. Cogn. 18, 820–822 10.1016/j.concog.2009.05.003 19625197

[B17] Ruz M. Moser A. Webster K. (2011). Social expectations bias decision-making in uncertain inter-personal situations. PLoS ONE 6, e15762 10.1371/journal.pone.0015762 21347404 PMC3036582

[B18] Ruz M. Tudela P. (2011). Emotional conflict in interpersonal interactions. Neuroimage 54, 1685–1691 10.1016/j.neuroimage.2010.08.039 20736070

[B19] Ruz M. Worden M. S. Tudela P. McCandliss B. D. (2005). Inattentional amnesia to words in a high attentional load task. J. Cogn. Neurosci. 17, 768–776 10.1162/0898929053747685 15904543

[B20] Sanfey A. G. (2009). Expectations and social decision-making: biasing effects of prior knowledge on ultimatum responses. Mind Soc. 8, 93–107 10.1007/s11299-009-0053-6

[B21] Sanfey A. G. Rilling J. K. Aronson J. A. Nystrom L. E. Cohen J. D. (2003). The neural basis of economic decision-making in the ultimatum game. Science 300, 1755–1758 10.1126/science.1082976 12805551

[B22] Schachter S. Hood D. Gerin W. Andreasson P. B. Rennert M. (1985). Some causes and consequences of dependence and independence in the stock market. J. Econ. Behav. Organ. 6, 339–357 10.1016/0167-2681(85)90003-4

[B23] Scharlemann J. P. W. Eckel C. C. Kacelnik A. Wilson R. K. (2001). The value of a smile: game theory with a human face. J. Econ. Psychol. 22, 617–640 10.1016/S0167-4870(01)00059-9

[B24] Schneider W. Eschman A. Zuccolotto A. (2002). E-prime User’s Guide. Pittsburgh: Psychology Software Tools Inc

[B25] Solnick S. (2001). Gender differences in the ultimatum game. Econ. Inq. 39, 189–200 10.1093/ei/39.2.189

[B26] Solnick S. J. Schweitzer M. (1999). The influence of physical attractiveness and gender on ultimatum game decisions. Organ. Behav. Hum. Decis. Process. 79, 199–215 10.1006/obhd.1999.2843 10471361

[B27] van’t Wout M. Kahn R. S. Sanfey A. G. Aleman A. (2006). Affective state and decision-making in the ultimatum game. Exp. Brain Res. 169, 564–568 10.1007/s00221-006-0346-5 16489438

